# The impact of disc degeneration on the dynamic characteristics of the lumbar spine: a finite element analysis

**DOI:** 10.3389/fbioe.2024.1384187

**Published:** 2024-05-01

**Authors:** Xue Wang, Wei Liu, Yaqiong Zhao, Pengcheng Ma

**Affiliations:** ^1^ The Sixth Affiliated Hospital of Xinjiang Medical University, Urumqi, China; ^2^ The Affiliated Tumor Hospital of Xinjiang Medical University, Urumqi, China; ^3^ Shandong Public Health Clinical Center, Shandong University, Jinan, China

**Keywords:** disc degeneration, lumbar spine, modal analysis, harmonic response analysis, time domain result

## Abstract

The dynamics of disc degeneration was analyzed to determine the effect of disc degeneration at the L4-L5 segment on the dynamic characteristics of the total lumbar spine. A three-dimensional nonlinear finite element model of the L1-S1 normal lumbar spine was constructed and validated. This normal model was then modified to construct two degeneration models with different degrees of degeneration (mild, moderate) at the L4-L5 level. Modal analysis, harmonic response analysis, and transient dynamics analysis were performed on the total lumbar spine when experiencing following compressive loading (500 N). As the degree of disc degeneration increased, the vibration patterns corresponding to the first three orders of the model’s intrinsic frequency were basically unchanged, with the first order being in the left-right lateral bending direction, the second order being in the forward-flexion and backward-extension direction, and the third order being in the axial stretching direction. The nucleus pulposus pressure peaks corresponding to the first order intrinsic frequency for the harmonic response analysis are all on the right side of the model, with sizes of 0.053 MPa, 0.061 MPa, and 0.036 MPa, respectively; the nucleus pulposus pressure peaks corresponding to the second order intrinsic frequency are all at the rear of the model, with sizes of 0.13 MPa, 0.087 MPa, and 0.11 MPa, respectively; and the nucleus pulposus pressure peaks corresponding to the third order intrinsic frequency are all at the front of the model, with sizes of 0.19 MPa, 0.22 MPa, and 0.22 MPa, respectively. The results of the transient analysis indicated that over time, the response curves of the healthy model, the mild model, and the moderate model all exhibited cyclic response characteristics. Intervertebral disc degeneration did not adversely affect the vibration characteristics of the entire lumbar spine system. Intervertebral disc degeneration significantly altered the dynamics of the degenerative segments and their neighboring normal segments. The process of disc degeneration gradually shifted the load from the nucleus pulposus to the annulus fibrosus when the entire lumbar spine was subjected to the same vibratory environment.

## 1 Introduction

Intervertebral disc degeneration is a progressive lesion that leads to changes in the geometry and biomechanical behavior of the disc, ultimately affecting its ability to transmit and distribute loads (Adams and Roughley, 2006). Lumbar disc degeneration is one of the most common causes of low back pain. Its causes are complex and multifactorial, including aging, abnormal mechanical loading and accidental injury (Luoma et al., 2000; Wilke et al., 2006; Diwan and Melrose, 2023). Early signs of disc degeneration include a decrease in the amount of water in the nucleus pulposus, which leads to a gradual change in the nucleus pulposus from a fluid to a solid state, as well as causing a decrease in disc height and swelling pressure (Urban and McMullin, 1988; Frobin et al., 2001; Benneker et al., 2005). As disc degeneration progresses, the increase in collagen in the nucleus pulposus leads to fibrosis, causing the nucleus pulposus to become rigid and the border between the annulus fibrosus and the nucleus pulposus to gradually blur (Adams, 2004; Inoue and Espinoza Orias, 2011). Changes in disc biochemistry and mechanical properties will inevitably affect spinal biomechanical properties.

For the lumbar spine, in a vibration environment, the stresses and strains in the lumbar spine tissues are equivalent to 2–3 times the size of the static load, and prolonged exposure to vibration may lead to degenerative changes in the lumbar joints of drivers. (Li et al., 2015; Taghizadeh-Alisaraei, 2017). Significant increase in disc stress and peak Von-Mises stress compared to normal disc (Li et al., 2017). Thus, overexposure to vibration is considered to be a major contributor to degenerative changes in the lumbar spine joints of drivers (Bovenzi and Betta, 1994; Jiang et al., 2021).

Numerous *in vitro* experimental studies have analyzed the effects of disc degeneration on the biomechanics of the spine, including the range of motion (ROM) of the lumbar spine (Mimura et al., 1994; Fujiwara et al., 2000a; Kettler et al., 2011), the small joint facet contact forces (Fujiwara et al., 2000b; Hicks et al., 2009), the intravertebral disc pressures (IDPs), and the distribution of stresses in the vertebral body (McNally and Adams, 1992; Johansson et al., 2017). For example, experimental studies by Mimura et al. (Mimura et al., 1994) and Kettler et al. (Kettler et al., 2011) found that the ROM of the degenerated segments during forward flexion-backward extension and left-right lateral bending decreases with increasing disc degeneration. Ruberne et al. (Ruberté et al., 2009) developed a finite element model of lumbar vertebrae L1-S1 with different degrees of degeneration in a single disc (L4-L5) and found that the peak Von-Mises stresses and tangential forces on the annulus fibrosus matrix of its adjacent segments during forward flexion-backward extension, right and left lateral bending, and axial torsion increase with increasing disc degeneration. However, these previous studies have mainly analyzed the effects of disc degeneration under static loading, and have not yet addressed the effects of disc degeneration on the biomechanical properties of the lumbar spine system in a vibration environment. Therefore, this study will focus on the effects of disc degeneration on the biomechanical properties of the lumbar spine system in a vibration environment.

## 2 Materials and methods

### 2.1 Geometric modeling

**FIGURE 1:**
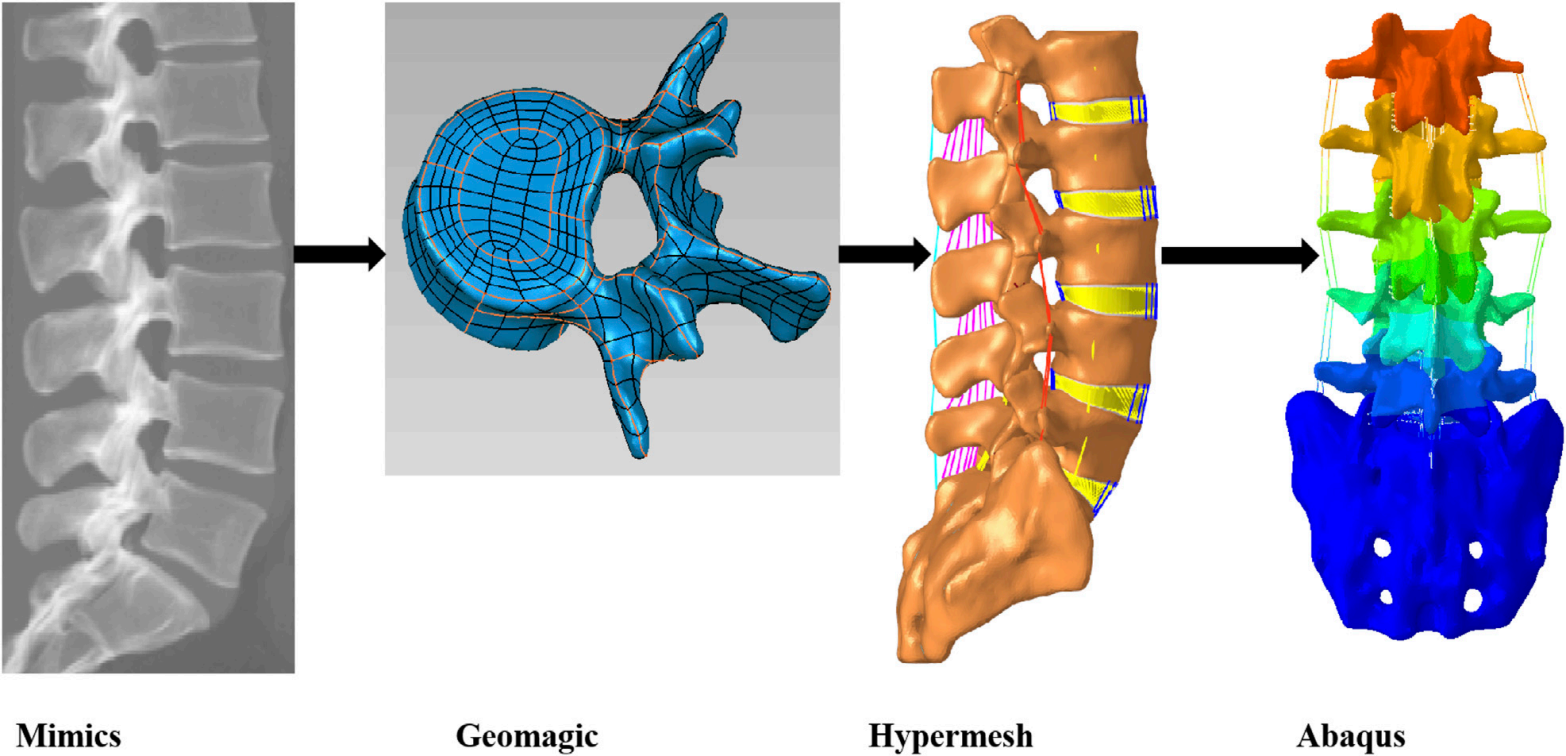
Technical roadmap for modeling the lumbar spine L1-S1 segments in healthy volunteers.

The volunteers for this study had detailed knowledge of the study and their written consent was obtained. The technical flow of this paper is shown in (Figure 1). A total of 511 DICOM images were acquired by CT from the upper edge of L1 to the sacrum with a slice spacing of 0.629 mm.Based on the CT scan dataset, a 3D geometric model of the lumbar spine was obtained by separating the target region and performing 3D reconstruction by setting a grayscale threshold and manually modifying it in Mimics 21.0 (Materialise, Belgium). The 3D model in Mimics software was exported in STL format to Geomagic Studio 12.0 (Geomagic, USA) for geometric routing and surface smoothing optimization, as well as reverse engineering to create the disc contour. Mesh delineation and material property assignment of the model was done in Hypermesh 2022 (Altair, USA), and finite element analysis calculations were performed using Abaqus 2021 (Simulia, USA) software.

### 2.2 Mesh delineation and material property assignment for finite element models

**FIGURE 2:**
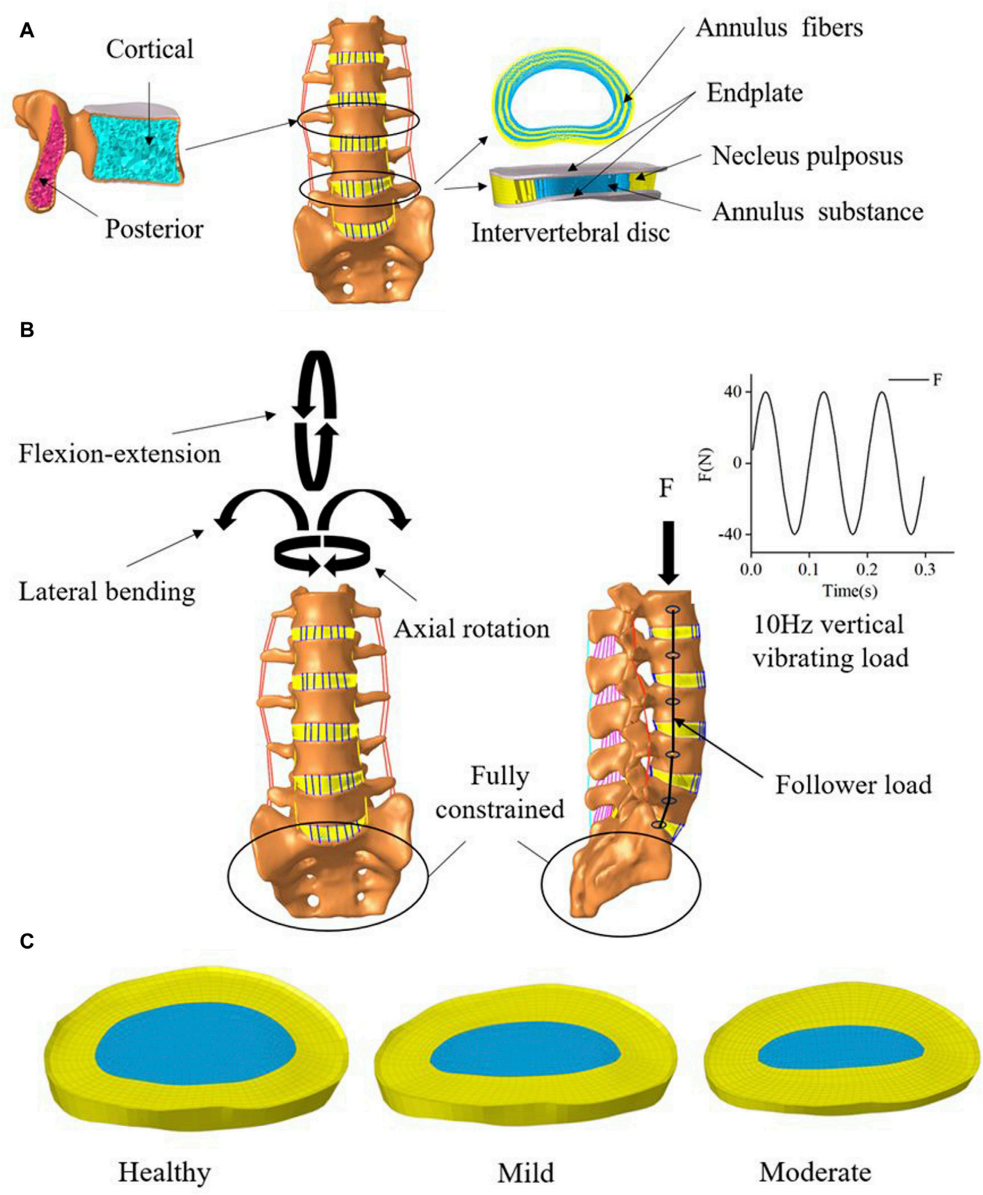
Mesh delineation and material property assignment for finite element models. **(A)** vertebral and Intervertebral disc model. **(B)** Boundary conditions **(C)** Intervertebral discs with 2 levels of degeneration.

The complete nonlinear finite element model shown in (Figure 2) includes cortical bone, cancellous bone, posterior structures, intervertebral discs, endplates, and ligaments. Cortical bone and cancellous bone are considered orthogonal anisotropic materials (Schmidt et al., 2007). Cortical bone thickness and endplate thickness were both set to 1 mm and defined as C3D8R elements (Li et al., 2023). The endplate mesh is connected to the vertebral body and the intervertebral disc mesh by a co-nodal method. The intervertebral disc consists of the nucleus pulposus, the fibrous matrix and the annulus fibrosus, with the nucleus pulposus accounting for approximately 44% of the disc volume (Sengupta, 2017). The fibrous ring consists of 6 layers of T3D2 elements of decreasing rigidity from lateral to medial (Table 1). The ligaments included seven ligament models of the capsular ligament (CL), intertransverse ligament (ITL), supraspinous ligament (SL), interspinous ligament (ISL), ligamentum flavum (LF), anterior longitudinal ligament (ALL), and posterior longitudinal ligament (PLL), and the ligament was modeled as a tensile-only truss (Polikeit et al., 2003).

**TABLE 1:** Material properties of the spinal components.

Spinal components	Young’s modulus (MPa)	Poisson’s ratio	Cross-sectional area ( ${mm}^{2}$ )	Element type	Density ${\left(10\right.}^{-6}kg/{mm}^{3}$ )
Cortical	$E_{xx}=11300$	$\upsilon_{xy}=0.484$		C3D8R (Hexahedral)	1.7
	$E_{yy}=11300$	$\upsilon_{yz}=0.203$			
	$E_{zz}=22000$	$\upsilon_{xz}=0.203$			
	$G_{xy}=3800$				
	$G_{yz}=5400$				
	$G_{xz}=5400$				
Cancellous bone	$E_{xx}=140$	$\upsilon_{xy}=0.450$		C3D4/C3D5(Tetrahedral/Pentahedral)	1.1
	$E_{yy}=140$	$\upsilon_{yz}=0.315$			
	$E_{zz}=200$	$\upsilon_{xz}=0.315$			
	$G_{xy}=48.3$				
	$G_{yz}=48.3$				
	$G_{xz}=48.3$				
Posterior	3,500	0.25		C3D4(Tetrahedral)	1.4
Endplate	24	0.4		C3D8R (Hexahedral)	1.2
Nucleus pulpous	C10 = 0.12, C01 = 0.03			C3D8RH(Hexahedral)	1.02
Annulus substance	C10 = 0.18, C01 = 0.045			C3D8RH(Hexahedral)	1.05
Annulus fibers				T3D2 (Truss)	
Outermost layer	550	0.3	0.7		1
Second layer	495	0.3	0.63		1
Third layer	440	0.3	0.55		1
Fourth layer	420	0.3	0.49		1
Fifth layer	385	0.3	0.41		1
Innermost layer	360	0.3	0.30		1
ALL	15		63.7	T3D2 (Truss)	1
PLL	18		20	T3D2 (Truss)	1
LF	12		40	T3D2 (Truss)	1
SSL	10		30	T3D2 (Truss)	1
ISL	10		40	T3D2 (Truss)	1
ITL	50		1.8	T3D2 (Truss)	1
CL	18		30	T3D2 (Truss)	1

### 2.3 Modeling of lumbar disc degeneration

In this study, On the basis of the established health model, two intervertebral discs with different degrees of degeneration were simulated. we modified two variables of disc morphology (disc height, nucleus pulposus area) and changed the material properties of the disc and adjacent endplates (Cai et al., 2020) as shown in Tables 2, 3. We constructed two models simulating mild and moderate lumbar degeneration at the L4-L5 level (Figure 2). The L4-L5 segment was chosen for disc degeneration simulation because the incidence of disc degeneration is higher in this segment (Ruberté et al., 2009).

**TABLE 2:** Changes in three variables of lumbar disc geometry morphology (Cai et al., 2020).

	Healthy	Mild	Moderate
Nucleus pulposus volume (%)	3,896 (45%)	2987 (35%)	1,278 (25%)
total volume	8,651	8,534	5,191
Proportion of intervertebral disc height reduction	0%	17%	50%

**TABLE 3:** Changes in material properties of lumbar intervertebral discs with different levels of degeneration (Cai et al., 2020).

	Healthy	Mild	Moderate
nucleus pulposus	C10 = 0.12, C01 = 0.03	C10 = 0.14, C01 = 0.035	C10 = 0.17, C01 = 0.041
matrix of fibrous rings	C10 = 0.18, C01 = 0.045	C10 = 0.18, C01 = 0.045	C10 = 0.18, C01 = 0.045
endplate	E = 24 MPa	E = 24 MPa	E = 100 MPa

### 2.4 Boundary conditions and contact settings

In this study, all the degrees of freedom at the bottom of S1 are fixed, a reference point is established at the upper surface of L1 and coupled to the upper surface node of L1. During the calibration and validation process, the ROM and the degree of deformation were calculated for pure torque loads (8 N-m flexural moment, 6 N-m transverse bending moment, 4 N-m rotational moment as in Figure 2) and 1200 N axial following loads. The accuracy of the normal model was verified by comparing the motions of each segment of the normal model with the previous sample data and finite element data (Renner et al., 2007; Park, Kim and Kim, 2013). In subsequent simulations, a following preload of 500 N was applied to the entire lumbar spine system (Figure 2) to simulate the upper body weight of the human body (Du et al., 2016). The model is then subjected to prestressed modal analysis, taking the first three orders of intrinsic frequency and mode of vibration below 30 Hz. Based on the modal analysis, the dynamic response of the healthy lumbar spine and disc degeneration lumbar spine under vibratory force excitation was further investigated. The modal steady-state dynamic analysis method was used to analyze the patient’s thoracolumbar sacral segment (L1-S1). Based on the modal analysis, the harmonic response analysis was performed using an equivalent damping ratio of 0.08. All degrees of freedom of the lower surface of the sacral vertebral body were fixed. A sinusoidal axial force of 40 N was applied to the upper surface of the vertebral body L1 with a frequency range of 0–30 Hz (Li et al., 2011). According to the modal analysis, the equivalent damping ratio for the transient analysis was also 0.08. All degrees of freedom of the lower surface of the sacral vertebral body were fixed. A sinusoidal axial force of 40N was applied to the upper surface of the vertebral body L1 (Figure 2) and the frequency was set to 10 Hz (Guo and Li, 2020).

## 3 Results

### 3.1 Model validation results

**FIGURE 3:**
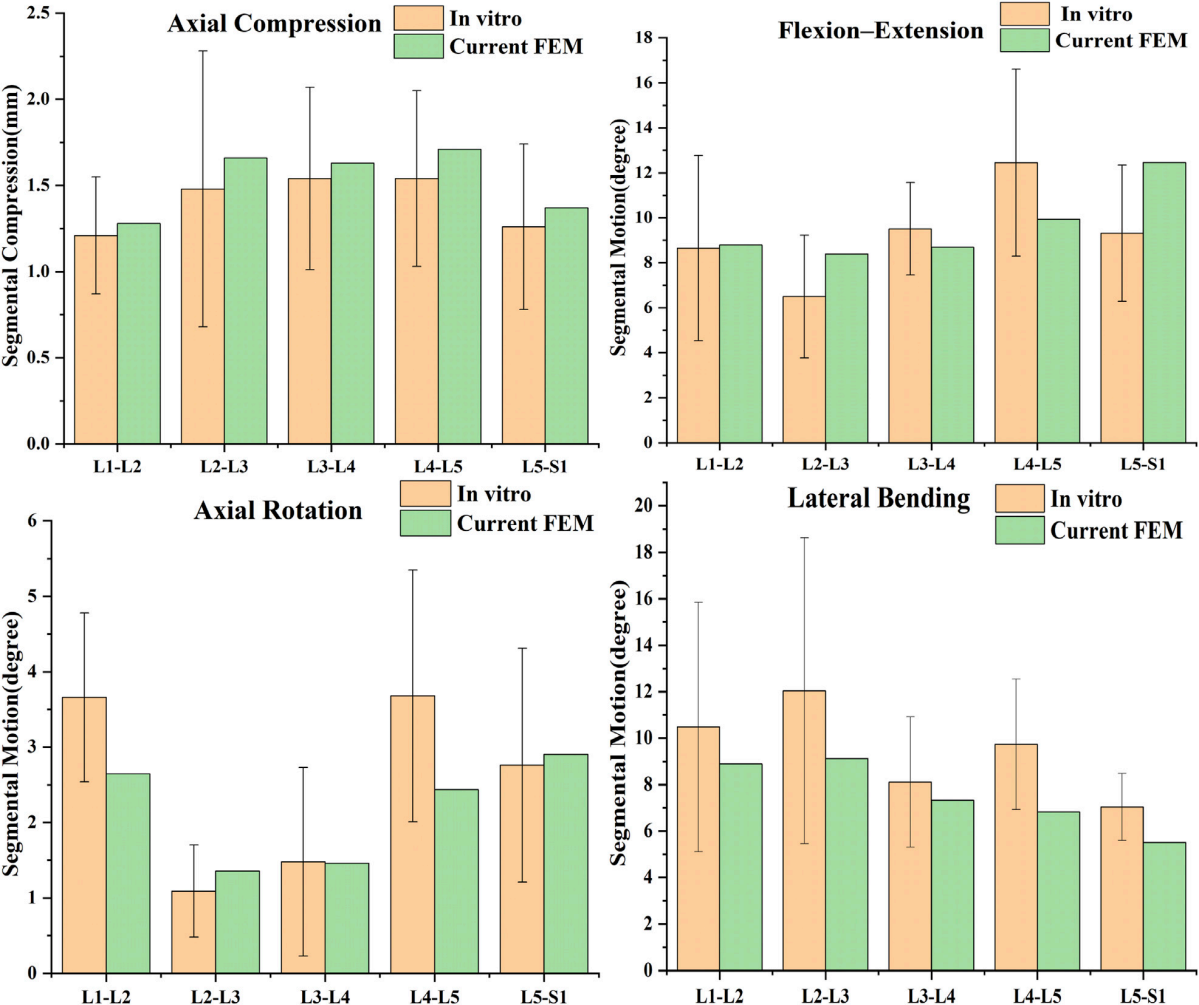
Comparison of predicted results by the current FE model with previous studies (Renner et al., 2007).

The current method of validating spine finite element models is mainly by comparing the ROM, mainly because the ROM of spine cadaveric specimens is easier to measure. As shown in the (Figure 3), the compression and ROM of each segment of the model are more consistent with Renner’s specimen experiment (Renner et al., 2007), which verifies the validity of the model.

### 3.2 Modal analysis results

**FIGURE 4:**
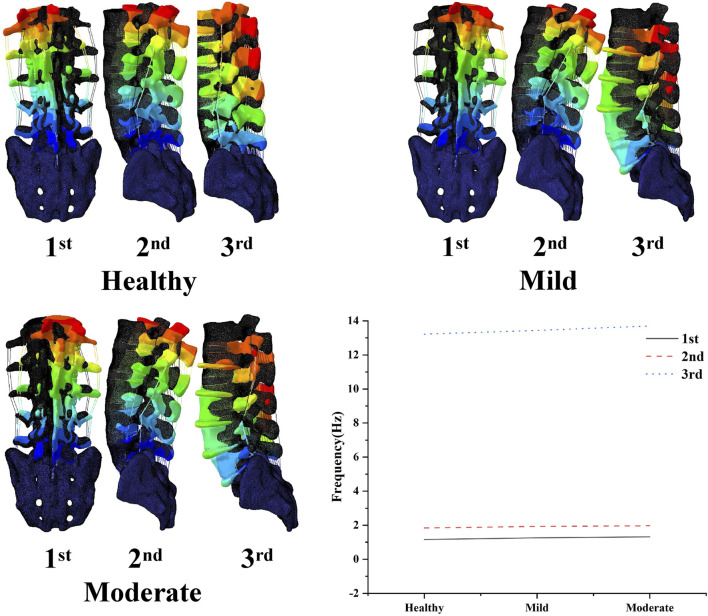
Variation of the first three orders of modal amplitude and resonance frequency of the lumbar spine model with different levels of degeneration.

The results of the modal analysis showed (Figure 4) that the first order intrinsic frequency of the model increased from 1.16 Hz to 1.26 Hz–1.32 Hz for the healthy model to the mildly degenerating disc model and the moderately degenerating model, the second order intrinsic frequency of the model increased from 1.84 Hz to 1.93 Hz–1.97 Hz, and the third order intrinsic frequency of the model increased from 13.22 Hz to 13.44 Hz–13.7 Hz, the vibration patterns corresponding to the first three orders of the model’s intrinsic frequency are basically unchanged, with the first order being in the left-right lateral bending direction, the second order being in the forward-backward direction, and the third order being in the vertical direction.

### 3.3 Harmonic response analysis results

**FIGURE 5:**
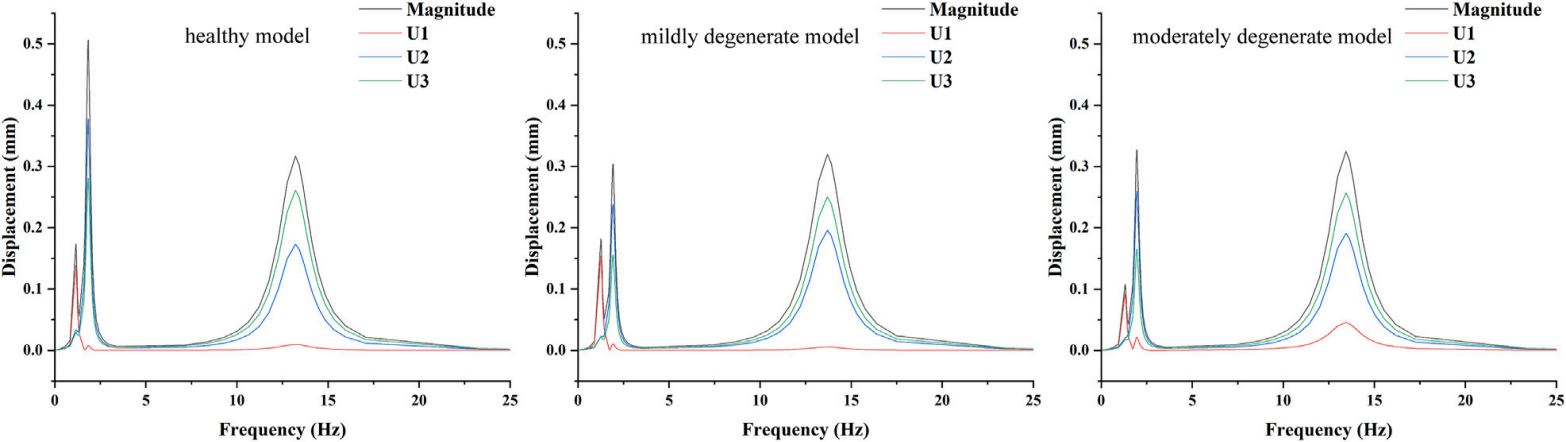
Mean displacement amplitude response curves of the nucleus pulposus fibrous ring at L4-L5 degenerative segments in lumbar spine models with different degrees of degeneration.

The results of the harmonic response analysis showed (Figure 5) that both the healthy and lumbar degeneration models showed varying degrees of wave crests at the displacement amplitudes corresponding to the first three orders of the intrinsic frequency, the displacement amplitude component of the healthy model at the first order intrinsic frequency is 0.14 mm maximum in the U1 direction, the displacement amplitude component at the second order intrinsic frequency has a maximum of 0.38 mm in the U2 direction, and the displacement amplitude component at the third order intrinsic frequency has a maximum of 0.26 mm in the U3 direction, the displacement amplitude component of the mildly degenerate model at the first order intrinsic frequency has a maximum of 0.15 mm in the U1 direction, the displacement amplitude component at the second order intrinsic frequency has a maximum of 0.24 mm in the U2 direction, and the displacement amplitude component at the third order intrinsic frequency has a maximum of 0.25 mm in the U3 direction, the displacement amplitude component of the moderately degenerate model at the first order intrinsic frequency has a maximum of 0.09 mm in the U1 direction, the displacement amplitude component at the second order intrinsic frequency has a maximum of 0.26 mm in the U2 direction, and the displacement amplitude component at the third order intrinsic frequency has a maximum of 0.26 mm in the U3 direction.

**FIGURE 6:**
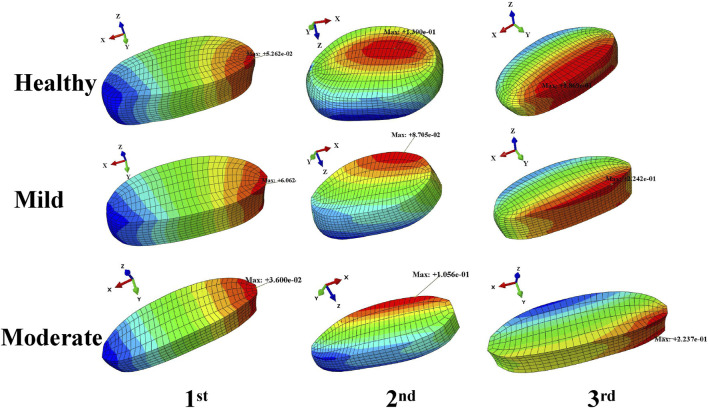
Distribution of medullary pressure at the peak of the displacement amplitude wave in the harmonic response analysis.

The disc pressures in the nucleus pulposus of the L4-L5 intervertebral disc corresponding to the three wave peaks in the harmonic response analysis are shown in (Figure 6), and the distribution of the amplitude of the disc pressures corresponding to the first three orders of the intrinsic frequency is basically the same for the healthy model and the mildly degenerated model and the moderately degenerated model, the maxima corresponding to the first order of the intrinsic frequency are to the right side of the model, with the sizes of 0.053 MPa, 0.061 MPa and 0.036 MPa; the maximum values corresponding to the second-order intrinsic frequency were all at the rear of the model, with sizes of 0.13 MPa, 0.087 MPa and 0.11 MPa; the maximum values corresponding to the third-order intrinsic frequency were all at the front of the model, with sizes of 0.19 MPa, 0.22 MPa and 0.22 MPa, respectively.

### 3.4 Transient analysis results

**FIGURE 7:**
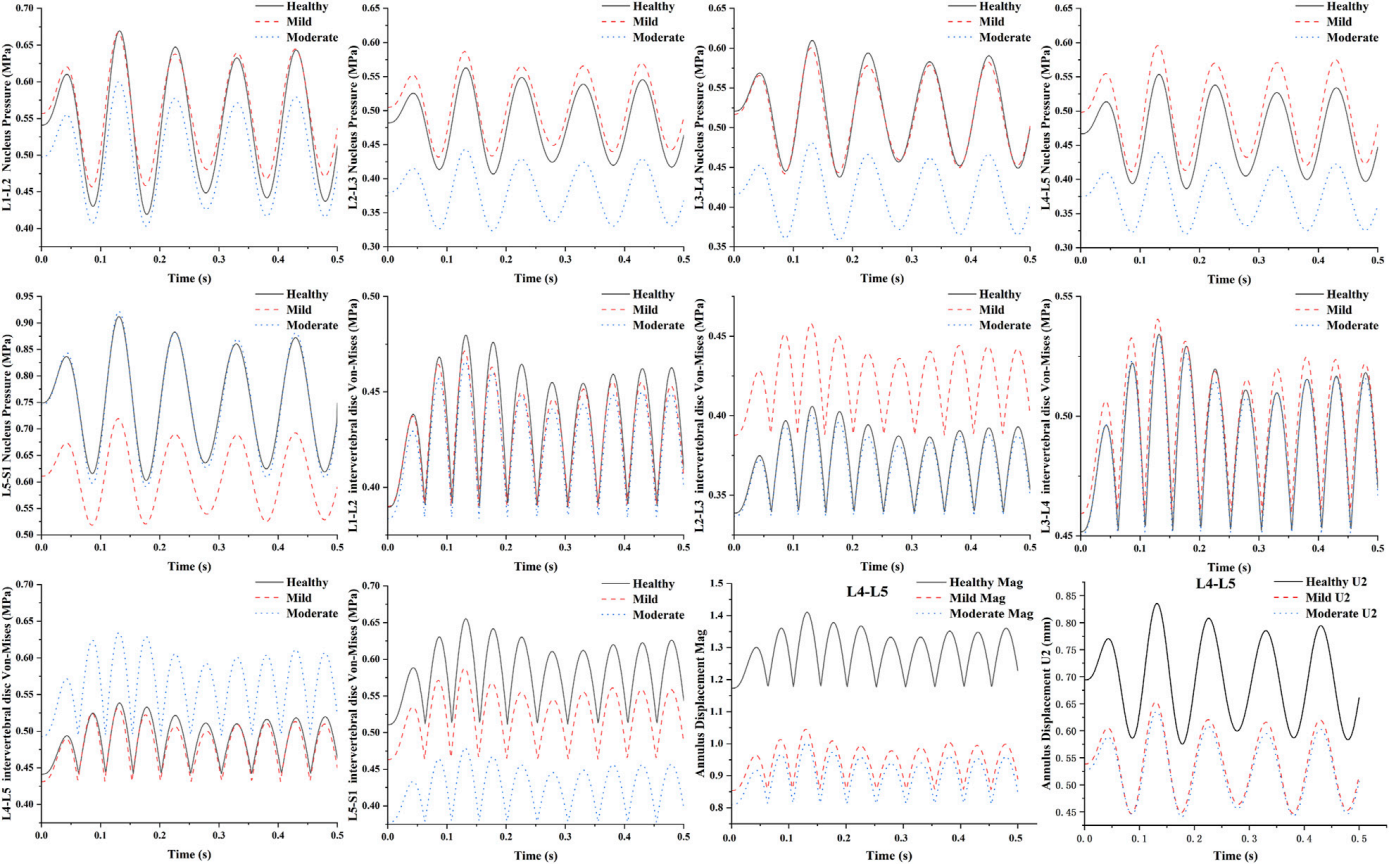
Time-domain dynamic response on L4-L5 lumbar segments with different degrees of degeneration under sinusoidal axial loading.

The results of the transient analysis indicated that the response curves of the healthy, mild degeneration, and moderate degeneration models exhibited cyclic response properties over time. The time-domain dynamic responses of all lumbar segments (L1-L2, L2-L3, L3-L4, L4-L5, and L5-S1) in L1-S1 for healthy, mild regression, and moderate regression as well as the results of the peak Von Mises stresses in the lumbar discs of L1-S1 under 10 Hz axial vibration loading are shown in (Figure 7). The results showed that the maximum values of the dynamic response curves of the nucleus pulposus in the L2-L3 and L4-L5 segments showed the same trend of change in the case of L4-L5 degeneration of the intervertebral discs, with the maximum pressure of the nucleus pulposus in the L2-L3 segment increasing from 0.56 MPa to 0.59 MPa and decreasing to 0.44 MPa, and that of the nucleus pulposus in the L4-L5 segment increasing from 0.55 MPa to 0.6 MPa and then decreased to 0.44 MPa, while the maximum pressure in the medulla in the L1-L2 and L3-L4 segments varied essentially the same, for example, the maximum pressure in the nucleus pulposus in the L1-L2 segment changed from 0.67 MPa to 0.67 MPa then decreased to 0.6 MPa. The peak Von-Mises stress in the L4-L5 intervertebral disc decreased from 0.54 MPa to 0.53 MPa and then increased to 0.63 MPa, and the peak Von-Mises stress in the healthy and mildly degenerated and moderately degenerated models of the L1-L2, L2-L3, and L3-L4 discs was basically unchanged, whereas the peak Von-Mises stress in the discs of the L5-S1 segments of the L5-S1 segment decreased sequentially from 0.66 MPa to 0.59 MPa–0.48 MPa. As the degree of deformation increases the degree of deformation of the fiber ring is decreasing in the anterior-posterior direction and the total displacement, the maximum displacement in the anterior-posterior direction decreases from 0.84 mm to 0.65 mm and then to 0.64 mm, and the total displacement decreases from 1.41 mm to 1.04 mm and finally to 1.0 mm.

## 4 Discussion

In this study, a finite element model of the normal lumbar spine at the L1-S1 level was constructed based on CT images. The validity of the model was verified by comparing the finite element simulation data with previously published sample experimental data and previous finite element experimental data. A finite element model of the lumbar spine with mild *versus* moderate degeneration was developed by varying the geometry and material properties of the intervertebral disc at the L4-L5 segment. Three levels of intervertebral disc models were included, which included the nucleus pulposus, the material properties of the annulus fibrosus matrix and endplates, and changes in disc height parameters were more consistent with the pathologic changes of advanced disc degeneration. Using these models, we evaluated the effects of disc degeneration on vibration in the lumbar spine system.

For the degenerated L4-L5 segment, as the degree of L4-L5 disc degeneration increased, the results of the modal analysis simulation showed that the increase in the degree of single-segment disc degeneration resulted in a small increase in the low-order intrinsic frequency of the whole lumbar spine system, but the corresponding vibration patterns remained basically unchanged, with the first order being in the left-right lateral bending direction, the second order being in the forward flexion and posterior extension direction, and the third order being in the axial stretching direction. Overall, disc degeneration did not lead to a deterioration of the vibration characteristics of the entire lumbar spine system (Guo and Fan, 2017).

The results of the harmonic response analysis showed that the displacement of the annulus fibrosus in the degenerated L4-L5 lumbar segments was mainly larger in the U2 direction, with the max U2 decreasing from 0.38 mm to 0.24 mm and then increasing to 0.261 mm. With the increase in the magnitude of the disc degeneration, the pressure in the nucleus pulposus of the L4-L5 increased slightly and then decreased markedly, and the distribution of the pressure in the nucleus pulposus was basically the same, which indicated that the increase in the degree of disc degeneration would not change the distribution of the pressure in the nucleus pulposus of the degenerated discs but would change its carrying capacity. The increase in the degree of disc degeneration will not change the distribution of pressure in the nucleus pulposus of the degenerated disc, but it will change its carrying capacity, and it will make the maximum pressure in the nucleus pulposus of the intervertebral discs slightly increase by slight degeneration of the intervertebral discs, but with the increase of the magnitude of the degeneration the maximum pressure in the nucleus pulposus significantly decreases.

In this study, the amplitude of the dynamic response of the L4-L5 intramedullary pressure decreased significantly as the degree of disc degeneration increased, while the L4-L5 maximum von Mises stress increased with the degree of disc degeneration, which is consistent with the results of previous hydrostatic studies (Cai et al., 2020; Wang, Li, Huang, Xu and Fan, 2023). This phenomenon suggests that the load-bearing capacity of the nucleus pulposus continues to diminish and the load borne by the annulus fibrosus continues to increase as the degeneration increases, i.e., the total load of the disc is shifted from the nucleus pulposus to the annulus fibrosus. At the same time, in the normal segment of L5-S1, the maximum von Mises stress and intramedullary pressure also decreased with the increase of disc degeneration. The amplitude of vibratory displacement of the annulus fibrosus also decreased, which is consistent with previous studies (Guo and Fan, 2018).

There are some limitations to this study, first, the structure of the human lumbar spine is extraordinarily complex, which leads us to some simplifications and constraints in finite element analysis. This study did not consider the near circular arc contour of intervertebral discs in the sagittal plane (Cappetti, Naddeo, Naddeo and Solitro, 2015). We acknowledge that our model does not incorporate the poroelastic behavior of the IVD, nor does it capture the permeability of nutrients within the disc (Elmasry et al., 2017; Elmasry et al., 2018; Volz et al., 2022). The whole lumbar spine finite element model in this paper was developed based on the CT data of a single volunteer, and the differences in geometric morphology between individuals may lead to differences in kinetic properties. Second, due to the difficulty of accurately modeling the true geometry of the ligaments, we simplified them to one-dimensional linear truss units, and the vertebrae were simplified to orthotropic anisotropic materials. But variations in vertebral bone density arise due to differences in age, gender, and other factors among individuals, which affecting the mechanical performance of vertebrae (Al-Barghouthi et al., 2020; Garay et al., 2022). And although we employed follower load techniques to approximate the effects of muscles and mitigate their absence on the biomechanical properties of the lumbar spine, we acknowledge that this method cannot fully substitute for the actual function of muscles. In addition, this study did not measure the specific values of intervertebral disc protrusion and did not quantify the reduction of intervertebral foramen in intervertebral disc degeneration (Amirouche et al., 2015). Despite these simplifications and limitations, the study in this paper shows consistency with previous studies.

## 5 Conclusion

In this study, we investigated the vibrational biomechanical properties of patients with disc degeneration in the vertical direction from a kinetic point of view. Our study showed that disc degeneration did not lead to the deterioration of the vibrational properties of the whole lumbar spine system, and that disc degeneration significantly altered the changes in the kinetic properties of the degenerative segments and their neighboring normal segments, and that during the process of disc degeneration when the whole lumbar vertebrae were in the same vibrational environment, the disc loads gradually shifts toward the annulus fibrosus.

## Data Availability

The original contributions presented in the study are included in the article/Supplementary material, further inquiries can be directed to the corresponding author.
